# Data set on impact of COVID-19 on mental health of internal migrant workers in India: Corona Virus Anxiety Scale (CAS) approach

**DOI:** 10.1016/j.dib.2021.107052

**Published:** 2021-04-17

**Authors:** Naveen Kumara Raghavendra, Suresh Gopal, Jothi Munuswamy

**Affiliations:** Department of Commerce, CHRIST (Deemed to be University), Bangalore 560029, India

**Keywords:** COVID-19, Corona Virus Anxiety Scale (CAS), Anxiety, Migrant workers, Mental health

## Abstract

The article presents a unique dataset on mental health of internal Migrant workers in India. The dataset was constructed during the pandemic when the entire nation was the victim of stringent measures to curtail the spread of Corona Virus in the form of travel restrictions and lockdowns. We collected this data in our pursuit to submit a paper in response to call for paper in the Journal titled “Migration and health”. Non-availability of authentic data about the internal migrant workers triggered this effort to compile the data. We have recorded 1350 responses out of a 6897 Sample through snowball sampling method. Every respondent is said to be a referee for further driving of sample. The responses were collected between June 2 and August 30, 2020 through the telephonic interviews. Also, the consent of the respondents has been duly obtained for publication of the data without revealing their identity. The interview schedule was adopted by using Corona virus Anxiety Scale (CAS) which uses four dimension model namely Cognitive, Emotional, Behavioral and Psychological. The Interview schedule was originally designed in English but was later translated into three different languages after consulting the language experts. This article provides descriptive statistics of study variables along with socio economic factors. This dataset provides a significant platform for further research related to CAS and in assessment of mental health of vulnerable groups.

## Specifications Table

**Table:** 

Subject	Social Science
Specific Subject area	Socio economics, Psychology
Type of Data	Tables Sav file xlsx file
How data was acquired	Telephonic Interview
Data format	Raw Analysed
Parameters of data collection	Respondents were chosen based on snowball sampling exclusively it was collected from internal migrant workers across India irrespective of the fact whether the location was affected by the pandemic or not.
Description of data collection	The survey was administered to internal migrant workers between June and August 2020 through telephonic interview from 1350 responses. Interview schedule is provided in the supplementary file.
Data source Location	Researchers from Christ University, Bangalore, have collected data from across India.
Data accessibility	Data Identification number: https://doi.org/10.17632/fkgf3fwnpz.1
	Direct URL to data: https://data.mendeley.com/datasets/fkgf3fwnpz/1

## Value of the Data

• This data is useful for researchers in measuring the levels of anxiety amongst the vulnerable group like migrant workers during uncertain times like pandemic.
• Enables researchers to carry out comparative studies on level of anxiety in normal and unusual situations.
• Provides insights on mental health conditions to carry out research on migrant workers in other countries.
• The data is useful to the policy makers to draft suitable policy reforms for welfare of vulnerable groups like migrant workers.

## 1 Data Description

Migrant workers in India are considered as the most vulnerable group to the pandemic [2]. They are vulnerable in terms of job security and economic condition [1]. As such, they are exposed to experience multiple psychological disorders like anxiety and depression. The data set is aimed at enhancing the knowledge of the users of the data on the impact of pandemic on the mental health issues of the internal migrant workers. Our survey probes into the psychiatric issues that migrant workers face and trauma that they underwent during the pandemic using Corona Virus Anxiety Scale (CAS) which was originally developed by Lee in the beginning of the year 2020. The scale uses four dimensions namely Cognitive, emotional, Behavioral and Psychological. The data was constructed with the help of interview schedule which was developed by employing Corona Virus Anxiety Scale (CAS). We have collected this data out of 1350 valid responses elicited through the telephonic interview. The data was collected during June to August 2020 which is considered to be the peak pandemic period. This data would enable the researcher to understand the possible adverse impacts on the anxiety levels of the dispersed population of migrant workers. The data set is provided in four tables. Table 1 gives the gist of questions that were asked during the telephonic interview and expected responses from the participants. Table 2 provides the details of descriptive statistics of Corona Virus Anxiety Scale (CAS) items with demographic factors. Mean value describes the mean score of particular type of respondents relating to the CAS items. For example from the Table 2, we can make out that the respondents whose age is up to 30 years registered mean score of 1.66 for the first CAS scale item. Thus, the mean is calculated with reference to each CAS scale item. Table 3 provides Migrants’ workers status on Corona Virus Anxiety scale items, Mean value describes that the average scores of the respondents to the CAS items. Table 4 provides Karl Pearson's Correlations coefficient between CAS dimensions and Coronavirus infection status. Negative correlation states that the respondents who were tested positive have more anxiety levels at all CAS dimensions namely cognitive, behavioral, emotional and physiological. Similarly, it also depicts that there is a positive correlation amongst different CAS dimensions as well. The interview schedule used for the data collection is given as a supplementary file. The raw data with coding is available in Mendeley Database for prospective researchers’ access.

**Table 1:** Interview Questions of the Telephonic Interview used for initial collection of information.

Questions	Possible Answers
What is your Age category?	Up to 30 Years old
	31-40 Years old
	41 & above
What is your Gender?	Male
	Female
What is your Martial Status?	Partnered
	Single
What is your level of Education?	No formal Education
	School dropout
	School completed
What is your Average Monthly Income?	Rs. 15000
	Rs. 15001-20000
	above Rs. 20000
Were you tested positive for Covid-19?	Yes, tested positive
	No, not tested positive
**How often have you experienced the following activities over the last 2 weeks?**	Not at all
Felt dizzy, lightheaded…..	Rare, less than a day
Lack of Sleep in…..	Several days
Felt paralyzed …..	More than a week
Lost interest in…..	Nearly every day over last 2 weeks”
Felt nauseous…..

**Table 2:** Descriptive statistics of Demographic and Corona Virus Anxiety factors.

							95% Confidence Interval for Mean			
Scale Item	Classification	N	Mean	Std. Deviation	Std. Error	Maximum	Lower Bound	Upper Bound	Minimum	
Dizziness	≤ 30 Years old	728	1.66	1.16	.043	4	1.58	1.75	0	
	31-40	345	1.60	1.11	.060	4	1.48	1.71	0	
	41 and Above	277	1.74	1.11	.067	4	1.61	1.88	0	
Lack of Sleep	≤ 30 Years old	728	1.67	1.22	.045	4	1.58	1.76	0	
	31-40	345	1.75	1.12	.060	4	1.63	1.87	0	
	41 and Above	277	1.77	1.15	.069	4	1.64	1.91	0	
Felt frozen	≤ 30 Years old	728	1.72	1.14	.042	4	1.63	1.80	0	
	31-40	345	1.81	1.11	.060	4	1.69	1.93	0	
	41 and Above	277	1.73	1.16	.070	4	1.60	1.87	0	
Loss of Interest in routine activities	≤ 30 Years old	728	1.67	1.24	.046	4	1.58	1.76	0	
	31-40	345	1.91	1.24	.067	4	1.78	2.04	0	
	41 and Above	277	1.82	1.19	.072	4	1.68	1.96	0	
Faced physical issues	≤ 30 Years old	728	1.61	1.18	.044	4	1.52	1.69	0	
	31-40	345	1.74	1.13	.061	4	1.63	1.86	0	
	41 and Above	277	1.65	1.10	.066	4	1.52	1.78	0	
Total score of CAS	≤ 30 Years old	728	8.33	4.40	0.16	18.00	8.01	8.65	0.00	
	31-40	345	8.82	4.20	0.23	17.00	8.37	9.26	0.00	
	41 and Above	277	8.71	4.04	0.24	17.00	8.24	9.20	0.00	
Dizziness	Male	758	1.59	1.17	.043	4	1.50	1.67	0	
	Female	592	1.76	1.09	.045	4	1.67	1.85	0	
Lack of Sleep	Male	758	1.60	1.22	.044	4	1.52	1.69	0	
	Female	592	1.85	1.12	.046	4	1.76	1.94	0	
Felt frozen	Male	758	1.63	1.15	.042	4	1.55	1.72	0	
	Female	592	1.89	1.10	.045	4	1.80	1.97	0	
Loss of Interest in routine activities	Male	758	1.70	1.25	.045	4	1.61	1.79	0	
	Female	592	1.84	1.22	.050	4	1.74	1.94	0	
Faced physical issues	Male	758	1.59	1.18	.043	4	1.51	1.67	0	
	Female	592	1.73	1.12	.046	4	1.64	1.82	0	
Total score of CAS	Male	758	8.11	4.54	.165	18.00	7.79	8.44	0.00	
	Female	592	9.08	3.86	0.16	18.00	8.76	9.39	0.00	
Dizziness	Married	823	1.77	1.11	.039	4	1.70	1.85	0	
	Unmarried	527	1.49	1.16	.051	4	1.39	1.59	0	
Lack of Sleep	Married	823	1.83	1.17	.041	4	1.75	1.91	0	
	Unmarried	527	1.53	1.18	.052	4	1.43	1.63	0	
Felt frozen	Married	823	1.85	1.16	.040	4	1.77	1.93	0	
	Unmarried	527	1.58	1.08	.047	4	1.49	1.67	0	
Loss of Interest in routine activities	Married	823	1.89	1.24	.043	4	1.80	1.97	0	
	Unmarried	527	1.57	1.21	.053	4	1.46	1.67	0	
Faced physical issues	Married	823	1.77	1.13	.039	4	1.69	1.84	0	
	Unmarried	527	1.47	1.16	.050	4	1.37	1.57	0	
Total score of CAS	Married	823	9.11	4.22	0.15	18.00	8.82	9.39	0.00	
	Unmarried	527	7.64	4.23	0.18	18.00	7.28	8.00	0.00	
Dizziness	Primary education	786	1.66	1.12	.040	4	1.58	1.74	0	
	Secondary Education	446	1.66	1.16	.055	4	1.56	1.77	0	
	No formal education	118	1.69	1.16	.107	4	1.47	1.90	0	
Lack of Sleep	Primary education	786	1.74	1.17	.042	4	1.66	1.82	0	
	Secondary Education	446	1.67	1.19	.057	4	1.56	1.79	0	
	No formal education	118	1.69	1.25	.115	4	1.46	1.91	0	
Felt frozen	Primary education	786	1.75	1.13	.040	4	1.67	1.82	0	
	Secondary Education	446	1.77	1.16	.055	4	1.66	1.87	0	
	No formal education	118	1.65	1.11	.102	4	1.45	1.86	0	
Loss of Interest in routine activities	Primary education	786	1.75	1.22	.044	4	1.66	1.83	0	
	Secondary Education	446	1.84	1.29	.061	4	1.72	1.96	0	
	No formal education	118	1.55	1.09	.100	4	1.35	1.75	0	
Faced physical issues	Primary education	786	1.64	1.16	.041	4	1.56	1.72	0	
	Secondary Education	446	1.67	1.13	.054	4	1.56	1.77	0	
	No formal education	118	1.65	1.18	.109	4	1.44	1.87	0	
Total score of CAS	Primary education	786	8.53	4.23	0.15	18.00	8.24	8.83	0.00	
	Secondary Education	446	8.61	4.36	0.21	18.00	8.20	9.02	0.00	
	No formal education	118	8.23	4.35	0.40	17.00	7.44	9.02	0.00	
Dizziness	up to 15000	415	1.46	1.18	.058	4	1.35	1.58	0	
	15000-20000	877	1.73	1.11	.038	4	1.66	1.80	0	
	> 20000	58	2.09	.99	.131	4	1.82	2.35	0	
Lack of Sleep	up to 15000	415	1.52	1.19	.059	4	1.41	1.64	0	
	15000-20000	877	1.77	1.18	.040	4	1.70	1.85	0	
	> 20000	58	2.19	.98	.129	4	1.93	2.45	0	
Felt frozen	up to 15000	415	1.52	1.09	.053	4	1.42	1.63	0	
	15000-20000	877	1.82	1.14	.039	4	1.74	1.90	0	
	> 20000	58	2.19	1.10	.144	4	1.90	2.48	0	
Loss of Interest in routine activities	up to 15000	415	1.48	1.18	.058	4	1.37	1.59	0	
	15000-20000	877	1.86	1.24	.042	4	1.77	1.94	0	
	> 20000	58	2.36	1.12	.147	4	2.07	2.66	0	
Faced physical issues	up to 15000	415	1.44	1.15	.056	4	1.33	1.55	0	
	15000-20000	877	1.72	1.14	.038	4	1.65	1.80	0	
	> 20000	58	2.07	1.15	.151	4	1.77	2.37	0	
Total score of CAS	up to 15000	415	7.43	4.22	.21	18.00	7.02	7.84	0.00	
	15000-20000	877	8.90	4.24	.14	18.00	8.62	9.18	0.00	
	> 20000	58	10.89	3.45	0.45	16.00	9.99	11.80	0.00

**Table 3:** Migrants’ workers status on Corona Virus Anxiety scale items.

					Mean		
Scale	N	Range	Minimum	Maximum	Statistic	Std. Error	Std. Deviation
Dizziness	1350	4.00	0.00	4.00	1.66	.031	1.14
Lack of Sleep	1350	4.00	0.00	4.00	1.71	.032	1.18
Felt frozen	1350	4.00	0.00	4.00	1.74	.031	1.14
Loss of Interest in routine activities	1350	4.00	0.00	4.00	1.76	.034	1.24
Faced physical issues	1350	4.00	0.00	4.00	1.65	.031	1.15
Total score of CAS	1350	18.00	0.00	18.00	8.53	0.12	4.28

**Table 4:** Correlations between CAS dimension and Coronavirus infection status.

	COVID Infection	Cognitive	Behaviour	Emotional	Physiological	Total score of CAS
COVID Infection	1					
Cognitive	−.386⁎⁎	1				
Behaviour	−.448⁎⁎	.577⁎⁎	1			
Emotional	−.345⁎⁎	.345⁎⁎	.489⁎⁎	1		
physiological	−.425⁎⁎	.440⁎⁎	.548⁎⁎	.507⁎⁎	1	
Total Score of CAS	−.512⁎⁎	.718⁎⁎	.810⁎⁎	.724⁎⁎	.860⁎⁎	1

⁎⁎ Correlation is significant at the 0.01 level (2-tailed).

## 2 Experimental Design, Materials and Methods

To construct this data set, the survey was conducted between June 2 and August 30, 2020, when the third level complete lock-down was exercised due to COVID 19. We initially identified 6897 internal migrant workers through a snowball sample method. To collect data, Interview schedule with translation in the local language was employed. A total of 1387 responses were obtained, however 1350 responses were considered for further analysis due to the elimination of invalid responses in the observations. As a matter of ethical concern, the identity of the respondents is kept confidential. Collected data were analysed with the help of SPSS software. The relationship between COVID infection status and anxiety levels was examined using Correlation Analysis. The interview schedule was prepared cautiously based on the literature reviews, which focuses on the socioeconomic status (SES) of the respondents and mainly on their anxiety level due to the COVID 19. Variables in the first part of the interview schedule were demographic and SES information, which includes age, gender, marital status, education status, income status and finally status of COVID 19. Variables in the second part were specifically designed based on Coronavirus Anxiety Scale - CAS [3] to collect and test the anxiety levels of internal migrant workers. That is, we adopted all five statements of CAS from Lee S.A. et al [3], which includes 1) I felt dizzy, lightheaded, when I read or listened to news about the coronavirus; 2). I had trouble falling asleep because I was thinking about the coronavirus; 3) I felt paralyzed or frozen when I thought about or was exposed to information about the coronavirus; 4) I lost interest in eating when I thought about or was exposed to information about the coronavirus and 5). I felt nauseous or had stomach problems when I thought about or was exposed to information about the coronavirus. All these variables were measured using CAS 5-point scale (0: not at all, 1: rare less than a day, 2: several days, 3: more than a week, 4: nearly every day over the last two weeks). The obtained data on CAS 5 statements were classified into four dimensions namely cognitive, behavioural, emotional and physiological for further analysis. A total of CAS score ≥9 indicates that there is a possibility of dysfunctional coronavirus related anxiety. High score in any of the individual CAS scale item or high total score of CAS reveals that problematic symptom for the person that might warrant further assessment or treatment.

## Ethics Statement

We, the authors, Naveen Kumara R, Suresh G and Jothi M, hereby declare that this dataset has not been previously published elsewhere nor this dataset is considered for publication elsewhere. Before collecting data from the respondents, the research objective was clearly explained to respondents. Consent of the respondents has been duly obtained for publication of the data without revealing their identity. We also obtained approval from the CHRIST (Deemed to be University), Bangalore, Research and Ethics Committee (REC). After reviewing the aim and methodology of the study, informed consent and interview schedule, the Committee has approved the data collection and has given ethics clearance vide number CU: RCEC/33/03/21 . Besides, research data confidentiality was communicated to the them, and was used exclusively for academic purposes.

## Declaration of Competing Interest

We as authors declare that we have no known competing financial interests or personal relationships, which have or could be perceived to have influenced our work reported in this article.

## Appendix Supplementary materials

Image, application 1
