# Supplementary material for: Data set on impact of COVID-19 on mental health of internal migrant workers in India: Corona Virus Anxiety Scale (CAS) approach
Source: Data Brief. 2021 Apr 17;36:107052. doi: 10.1016/j.dib.2021.107052 (PMC8114112; doi:10.1016/j.dib.2021.107052)
Supplement: Supplementary file 1 [file mmc1.docx]

APPENDIX 1

INTERVIEW SCHEDULE

1. Age : Up to 30 yrs old 31 – 40 yrs old 41 and above
2. Gender : Male Female
3. Marital status :Married Unmarried
4. Education status :Primary Secondary No formal education
5. Income status(Rs. p.m.) :up to Rs.15000 15001 to 20,000 Above 20000
6. status of COVID 19 : Affected Not Affected

7. How often have you experienced the following activities over the last 2 weeks ?

*(Interviewer shall record the responses personally in the scoring sheet).*

|  |
| --- |

| QN | Statement | 0 | 1 | 2 | 3 | 4 |
| --- | --- | --- | --- | --- | --- | --- |
| 1 | Felt dizzy, lightheaded, or faint, when read or listened to news about the coronavirus. |  |  |  |  |  |
| 2 | Faced trouble in falling asleep thinking about the corona virus |  |  |  |  |  |
| 3 | Felt paralyzed or frozen when thought about corona virus |  |  |  |  |  |
| 4 | Lost interest in eating while thinking about Corona Virus |  |  |  |  |  |
| 5 | Felt nauseous or had stomach problems thinking about Corona Virus or when exposed to information about the Corona Virus |  |  |  |  |  |

Note: 0 - not at all, 1 - rare less than a day, 2 - several days, 3 - more than a week, 4 - nearly every day over the last two weeks

| APPENDIX 2  ***Coding Sheet for the Interviewer*** | | | | | | |
| --- | --- | --- | --- | --- | --- | --- |
| ***Respondent Number : Interviewer Name:*** | | | | | | |
|  | ***Interviewer records the response through a tick mark in appropriate column to the questions*** | | | | | |
| Code | Questions | 1 | 2 | 3 | 4 | 5 |
| **Age** | What is your Age category? |  |  |  |  |  |
| **Gender** | What is your Gender? |  |  |  |  |  |
| **Marital status** | What is your Martial Status? |  |  |  |  |  |
| **Education** | What is your level of Education? |  |  |  |  |  |
| Income | What is your Average Monthly Income? |  |  |  |  |  |
| **Covid- infected or not** | were you tested positive for Covid-19? |  |  |  |  |  |
|  | How often have you experienced the following activities over the last 2 weeks? |  |  |  |  |  |
| CAS1 | Felt dizzy, lightheaded, or faint, when read or listened to news about the coronavirus. |  |  |  |  |  |
| CAS2 | Faced trouble in falling asleep thinking about the corona virus |  |  |  |  |  |
| CAS3 | Felt paralyzed or frozen when thought about corona virus |  |  |  |  |  |
| CAS4 | Lost interest in eating when I thought about Corona Virus |  |  |  |  |  |
| CAS5 | Felt nauseous or had stomach problems when you thinking about Corona Virus or when I was exposed to information about the Corona Virus |  |  |  |  |  |
